# Effectiveness and Safety of *Panax ginseng* Extract on Hepatic Dysfunction: A Randomized, Double-Blind, Placebo-Controlled Clinical Trial

**DOI:** 10.1155/2020/2689565

**Published:** 2020-07-16

**Authors:** Lei Shen, Si Ra Gwak, Jong Cheon Joo, Bong Keun Song, Seon Woo Cha, Young Uk Song, Mi Kyung Pyo, Soo Jung Park

**Affiliations:** ^1^Department of Constitutional Medicine, College of Korean Medicine, Wonkwang University, Iksan 54538, Republic of Korea; ^2^Department of Internal Medicine, College of Korean Medicine, Wonkwang University, Gwangju 51729, Republic of Korea; ^3^International Ginseng and Herb Research Institute, Geumsan 32724, Republic of Korea; ^4^Daedong Korea Ginseng Co., Ltd., Geumsan 32718, Republic of Korea; ^5^Department of Sasang Constitutional Medicine, College of Korean Medicine, Woosuk University, Jeonju 55338, Republic of Korea

## Abstract

**Background:**

The purpose of this study was to evaluate the efficacy and safety of *Panax ginseng* extract (GS-KG9) in the treatment of hepatic dysfunction.

**Methods:**

A randomized, double-blind, placebo-controlled clinical trial was conducted from December 2017 to January 2019. The trial included 60 subjects between the ages of 19 and 70 who had higher alanine transaminase (ALT) levels than the normal upper limit. The subjects were randomly divided into two groups: GS-KG9 (*n* = 30) and placebo (*n* = 30). The former was administered three GS-KG9 capsules (3 g/day) and the latter three placebo capsules (3 g/day) twice each day orally after meals in the morning and evening for 12 weeks. The primary goal was to observe the changes in ALT and gamma-glutamyl transferase (GGT) levels. The safety of the treatment was assessed and adverse events (AEs) were recorded.

**Results:**

Out of 60 subjects, nine were excluded from the efficacy analysis because they met the exclusion criteria. Therefore, a total of 51 subjects were evaluated for the effectiveness of the treatment (26 in the GS-KG9 group and 25 in the placebo group). After 12 weeks of treatment, the ALT levels were significantly reduced in the GS-KG9 group compared to the placebo group (*p*=0.009). The GGT level of the GS-KG9 group was significantly lower than that of the placebo group (*p*=0.036). Mild AEs, such as diarrhea, occurred during the study. There were no significant differences between the two groups.

**Conclusion:**

The results of this trial suggest that GS-KG9 might be an effective and safe option for mild hepatic dysfunction. This trial is registered with KCT0004080.

## 1. Introduction

The liver is the largest gland organ in the body; it mainly regulates the metabolism of fats, carbohydrates, and proteins and participates in the secretion and excretion of bile [1, 2]. When hepatic function is damaged, it often leads to abnormal metabolism and sometimes obstructive diseases. However, early hepatic dysfunction has no clear clinical symptoms, so it is often neglected, leading to further liver damage and liver disease. At present, conventional drugs for treating liver diseases caused by hepatic dysfunction are inadequate, and may have some side effects, so it is important to identify safe and effective complementary and alternative therapies [3]. Natural plant-derived herbs have been used in clinics for many years. It has been reported that a variety of herbs are effective in the treatment of hepatic dysfunction, with few side effects [4, 5]. Therefore, the prospect of using herbs to develop hepatoprotection is very promising.

Ginseng (*Panax ginseng* C.A. Meyer) is a perennial herb in the Araliaceae family. It is a traditional medicinal plant that has been used in clinics for a long time. In previous reports, ginseng extracts have been shown to protect the liver, offer immunomodulatory effects, and have no significant adverse effects [6, 7]. People have a good tolerance for ginseng. A systematic review on the safety of using ginseng showed that the frequency or symptoms of ginseng AEs were not significantly different from those of the placebo, and there were no severe adverse events [8]. Another systematic review also concluded that ginseng is safe to use [9].

According to reports, ginseng has a protective effect on hepatic dysfunction in various animals. *In vivo* studies have shown that ginseng extract has protective and preventive effects on a variety of hepatotoxins, such as carbon tetrachloride (CCl_4_), d-galactosamine (GalN), cyclophosphamide (CP), alcohol, and paracetamol (acetaminophen) [10–12]. Ginseng has also been shown to have antidepressant-like effects [13].

Ginsenoside is the main active ingredient in ginseng and has many physiological and pharmacological uses, e.g., anti-inflammation, antioxidation, and antifatigue [14]. Studies have shown that Rg1 can decrease the production of inflammatory cytokines and reduce the inflammation in the liver [15]. Rb1 may reduce liver fat accumulation in obese mice by mediating an upregulation of perilipin expression in adipocytes [16]. Rb2 can alleviate liver lipid accumulation by inducing Sirt1 and activating AMP-activated protein kinase (AMPK) to restore autophagy [17].

Few studies have explored the efficacy and safety of *Panax ginseng* extract (GS-KG9) on hepatic dysfunction in humans, and this study aimed to scientifically evaluate GS-KG9 in this context. This will provide a good basis for future studies.

## 2. Materials and Methods

### 2.1. Participants

This study conducted a 12-week, multicenter, randomized, double-blind, placebo-controlled clinical trial to evaluate the efficacy and safety of GS-KG9 on hepatic dysfunction. All subjects had to meet the following inclusion criteria: (1) aged 19 to 70 years; (2) having higher ALT levels than the normal upper limit of the each institution; (3) giving written informed consent. Exclusion criteria were (1) having threefold the normal levels of ALT; (2) having taken medications or dietary supplements that influenced hepatic function within four weeks prior to screening; (3) having taken antipsychotics within two months prior to screening; (4) having a history of substance or alcohol abuse; (5) being allergic to the ingredients in ginseng or red ginseng; (6) having a history of disease that could interfere with the test products or impede their absorption, such as gastrointestinal diseases (Crohn's Disease) or gastrointestinal surgery (except appendectomy and enterocele surgery); (7) having hepatitis B virus (HBV) or hepatitis C virus (HCV), cirrhosis, or liver cancer; (8) being a hepatitis carrier; (9) having a history of acute/chronic diseases, including cardiovascular, cerebrovascular, endocrine, immunologic, respiratory, hepatobiliary, kidney, urologic, neurological or psychiatric, musculoskeletal, inflammatory, hematologic, and neoplastic diseases; (10) reporting high alcohol intake (>21 units/week) within three months prior to screening; (11) having creatinine levels > 2.0 mg/dL; (12) being pregnant or breastfeeding; (13) having participated in other clinical trials within two months prior to screening; (14) having taken herbal supplements within four weeks prior to taking study medication for the first time; (15) women of childbearing age not adhering to acceptable forms of contraception; (16) having a history of more than one of diseases such as esophageal variceal bleeding, hepatic encephalopathy, and ascites within one year prior to taking study medication for the first time.

### 2.2. Study Design

**Figure 1:**
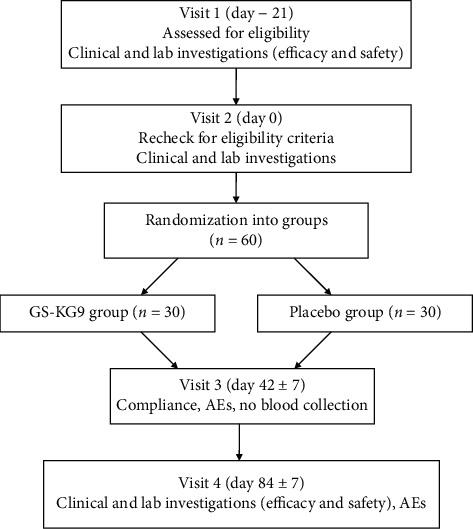
Systemic illustration of the study design. GS-KG9: *Panax ginseng* extract; AEs: adverse events.

This was a multicenter study (Wonkwang University Gwangju Medical Center, Gwangju; Wonkwang University Oriental Medical Hospital, Jeonju) in the Republic of Korea from December 2017 to January 2019. Subjects were recruited through online and offline advertising. There were a total of four visits (Figure 1): visit 1, screening (day 21); visit 2, randomization (day 0); visit 3, follow-up (day 42 ± 7 days); visit 4, end of the study (day 84 ± 7 days). The principal investigator initially screened each potential subject against the inclusion and exclusion criteria. All subjects were screened and evaluated for hepatic function test parameters (ALT, GGT, aspartate transaminase (AST), alkaline phosphatase (ALP), total bilirubin, total protein, serum albumin), lipid profiles (total cholesterol (TC), triglyceride (TG), high-density lipoprotein cholesterol (HDL-C), and low-density lipoprotein cholesterol (LDL-C)), highly sensitive-C reactive protein (hs-CRP), viral hepatitis test (HBV surface antigen and HCV antibody), and hepatic ultrasonography; subjects also underwent an electrocardiogram (ECG) and pregnancy test. The assessments of physical activity and dietary intake were performed at baseline and every 6-week visit. At each visit, all participants were asked to submit a diary in which they recorded all food intake for at least 3 days (including 1 weekend day). Subjects provided the physical activity data using the Global Physical Activity Questionnaire (GPAQ) at each visit. A clinical research coordinator (CRC) reviewed all the diaries and provided the participants with counseling on diet and physical activity. All subjects who met the inclusion criteria agreed to maintain their typical diet and exercise habits. In addition, all eligible subjects signed a written informed consent form before enrollment. The enrolled subjects were randomly assigned to one of two groups: GS-KG9 (*n* = 30) or placebo (*n* = 30).

The study was conducted according to the Declaration of Helsinki and Korean Good Clinical Practice guidelines. This study followed the CONSORT guidelines for randomized clinical trials. It was approved by the Institutional Review Board (IRB) of the Wonkwang University Korean Medical Hospital in Jeonju (WUJKMH-IRB-2017-0003) and Gwangju (WUJKMH-IRB-2017-0012). This study was registered with the Korean Clinical Trial Registry (CRIS), Republic of Korea, KCT0004080.

### 2.3. Randomization

A randomization scheme was generated by a computerized procedure. Enrolled participants were assigned to one of the two study groups (GS-KG9 or placebo) according to a computer-generated randomization sheet in a 1 : 1 ratio. The allocation sequence was concealed from the researcher enrolling and assessing participants in sequentially numbered, opaque, sealed, and stapled envelopes. The randomization sequence and allocation results were concealed to all subjects, research staff, investigators, and pharmacists until the final data were obtained. The master randomization list with the details on allocation was kept safely and confidentially with the study sponsor.

### 2.4. Study Medication

GS-KG9 made by Daedong Korea Ginseng Co., Ltd. (Geumsan, Republic of Korea) was obtained from International Ginseng & Herb Research Institute (Geumsan, Republic of Korea). The ginseng was dried, then extracted twice with 70% ethyl alcohol solution at 40° vacuum condition, and concentrated under reduced pressure and lyophilized.

The content of ginsenosides Rg1 and Rb1 in the ginseng extract was 12 ± 2.4 mg/g. Marker compounds were detected by high-performance liquid chromatography at a wavelength of 203 nm. Sixty subjects were randomly assigned to the GS-KG9 group (*n* = 30) and the placebo group (*n* = 30). The duration of treatment was 12 weeks, with either GS-KG9 3.0 g (GS-KG9, 2.4 g/day) or placebo 3.0 g (GS-KG9, 0 g/day), both administered as three capsules, twice a day, orally after meals. The GS-KG9 capsule consisted of 80% GS-KG9, 18% crystalline cellulose, 1% magnesium stearate, and 1% silicon dioxide. The placebo capsule consisted of 90% crystalline cellulose, 4% ginseng flavor powder, 4% caramel coloring, 1% magnesium stearate, and 1% silicon dioxide. Ginseng and the placebo capsules had the same taste and appearance (Table 1).

### 2.5. Outcome Measures

The primary outcomes were determined based on the levels of ALT and GGT; the secondary outcomes were determined based on levels of AST, ALP, and total bilirubin. The safety of the treatment was evaluated by laboratory tests; measures of vital signs, ECG, AEs; and a physical examination. Blood samples were taken after 12 hours of fasting. Samples were determined using the autoanalyzers Hitachi 7180 (Hitachi, Tokyo, Japan) in Jeonju and Hitachi 7020 (Hitachi, Tokyo, Japan) in Gwangju. The clinical laboratory parameters and ECG were measured before and after the 12-week treatment. Compliance (assessed by the number of returned capsules) and AEs were assessed during the follow-up (visit 3) and at the end of the study (visit 4). Vital signs, smoking status, alcohol intake, physical activity, and dietary intake were measured every six weeks.

### 2.6. Sample Size

The sample size was calculated based on a pilot study that compared serum ALT levels of the test and placebo groups [18]. We assumed that, after 12 weeks of treatment, changes in ALT levels would be 14.4 IU/L in the GS-KG9 group and 4.3 IU/L in the placebo group, with a standard deviation of 14 IU/L, statistical power of 80%, and alpha set of 5%. We set the total sample size to 60 (30 for each group) and estimated a dropout rate of 20%.

### 2.7. Statistical Analysis

Statistical analysis was performed using SAS version 9.3 for Windows. *p* < 0.05 was considered statistically significant. All data were entered into a data sheet twice and reviewed to ensure accuracy. The assessments of efficacy outcomes were primarily based on the full analysis set (FAS), and the variables were expressed as mean ± standard deviation (SD). Baseline characteristics were compared using a Chi-square test or Fisher's exact test and independent *t*-test. When statistically significant differences between groups were found in baseline characteristics, especially age and sex, they were used as covariates for efficacy analysis. ANCOVA was used for statistical analysis of covariates of baseline characteristics. Differences between the two groups were compared using an independent *t*-test. For the safety assessment, the Chi-square test or Fisher's exact test was conducted to evaluate the differences in the prevalence of adverse events between the two groups.

## 3. Results

### 3.1. Study Population and Baseline Data

**Figure 2:**
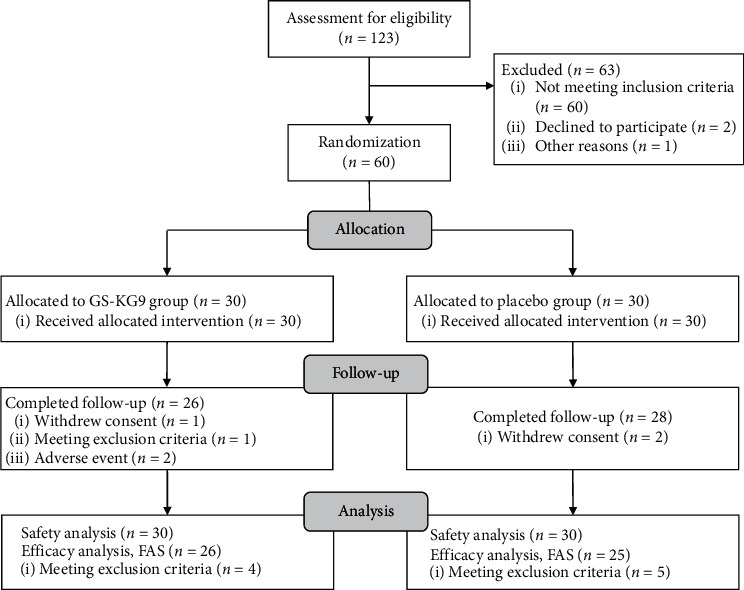
Flowchart of the study subjects. GS-KG9: *Panax ginseng* extract; FAS: full analysis set.

The flowchart of the clinical trial is shown in Figure 2. Among the 60 randomized subjects, 9 were excluded from the efficacy analysis: two cases of threefold higher-than-normal levels of GGT (*n* = 2 for placebo), five cases of gallstones (*n* = 3 for GS-KG9, *n* = 2 for placebo), and two cases of diabetes mellitus (*n* = 1 for GS-KG9, *n* = 1 for placebo). Thus, 51 subjects were included in the FAS analysis (*n* = 26 for GS-KG9, *n* = 25 for placebo). Four subjects in the GS-KG9 group dropped out after enrollment (one withdrew consent; one met exclusion criteria; and two withdrew due to AEs) and so did two in the placebo group (they withdrew consent). Therefore, 54 subjects completed this study. The safety analysis was performed on 30 subjects in the GS-KG9 group and 30 in the placebo group. The compliance rates of the GS-KG9 and placebo groups were 92.02 ± 6.85% and 92.90 ± 4.90%, respectively.

Baseline characteristics are shown in Table 2. Age, sex, waist circumference, weight, smoking, alcohol consumption, and blood pressure were not significantly different between groups at the baseline (*p* > 0.05). Body mass index (BMI) and alcohol consumption remained stable throughout the study period in both groups. Total bilirubin in the GS-KG9 group was lower than in the placebo in the baseline of efficacy evaluation (*p*=0.043). There was no significant difference between the two groups in other variables (*p* > 0.05).

### 3.2. Efficacy

**Figure 3:**
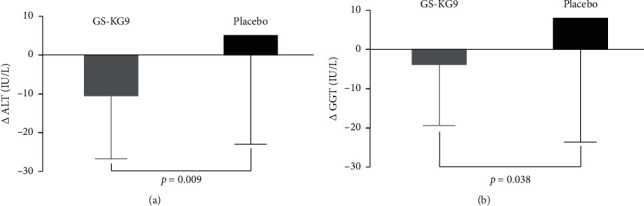
(a) The changes of ALT level at baseline (0 week) and after 12 weeks in the GS-KG9 and placebo groups (*p*=0.009 between the groups). (b) The changes of GGT level at baseline (0 week) and after 12 weeks in the GS-KG9 and placebo groups (*p*=0.036 between the groups). GS-KG9: *Panax ginseng* extract; ALT: alanine transaminase; GGT: gamma-glutamyl transferase.

The changes in ALT and GGT levels in the GS-KG9 and placebo groups are shown in Figure 3. GS-KG9 treatment was associated with decreased levels of ALT and GGT. Compared to the placebo, ALT and GGT levels in the GS-KG9 group significantly decreased over the 12 weeks.

The changes in hepatic function (ALT, GGT, AST, ALP, and total bilirubin) in the GS-KG9 and placebo groups are shown in Table 3. Compared to the placebo group, ALT and GGT levels significantly decreased in the GS-KG9 group after 12 weeks (*p*=0.009, *p*=0.036). The changes in AST, ALP, and total bilirubin levels were not statistically different between the two groups (*p* > 0.05).

### 3.3. Safety

The results of the laboratory tests (hematology and biochemistry) at the baseline and after the 12 weeks of treatment are shown in Table 4. There were no significant differences in blood chemistry parameters between the two groups after the 12 weeks. We also observed dietary intake and physical activity. Overall, there were no significant differences between the two groups for any parameter (calories, protein, carbohydrate, and dietary fiber) except dietary intake of lipids (data not shown). There were no significant differences between the two groups in physical activity (data not shown).

There were four serious adverse events (SAEs) reported during the study period. The SAEs included one case of a rupture in the cervical disc and one case of influenza virus A in the GS-KG9 group, and one case of lower back pain and one case of pain at the ankle and back in the placebo group. There were 20 AEs, 19 of which were mild and one of which was moderate. The mild AEs included two cases of indigestion, one case of upper respiratory tract infection, one case of abdominal inflation, one case of diarrhea, one case of rash, one case of chest pain, one case of chest discomfort, one case of left thigh numbness, one case of cervical pain, and one case of elevated creatine level in the GS-KG9 group; two cases of cold, one case of reflux esophagitis, one case of enteritis, one case of heartburn, one case of platelet reduction, one case of nausea, and one case of left ear infection in the placebo group. One moderate AE was an increase in AST and ALT levels in the placebo group.

SAEs in the GS-KG9 and placebo groups were reported, and their irrelevance to this study was approved by IRB. Among the mild AEs, one case of diarrhea occurred in the GS-KG9 group, which may be related to the intake of the study medication. Therefore, the subject stopped taking the study medication and dropped out of the study. However, the subject recovered completely after 20 days and no longer felt any discomfort. All other AEs were judged to be unrelated to the intake of the study medication. There were no significant differences in AEs between the two groups (*p*=0.775).

## 4. Discussion

Compared to the control group, the levels of serum ALT and GGT were significantly reduced after 12 weeks of GS-KG9 intake. An increased level of ALT indicates that damage has occurred to hepatic function. ALT may be useful as a screening test for the early detection of asymptomatic liver diseases [19]. GGT is a cell surface enzyme [20] that mainly exists in the cell membrane of organs such as the liver, pancreas, spleen, kidney, heart, and brain. Damaged liver parenchyma can lead to an increase in GGT levels. The reduced levels of ALT and GGT indicate that the GS-KG9 treatment improved hepatic function.

Ginseng extract has been reported to have many functions, such as antioxidation, anti-inflammation, and antifatigue [21, 22]. The main active ingredient in ginseng, ginsenoside Rg1, has protective effects on D-gal-induced liver injury in mice by inhibiting oxidative stress [23]. *In vitro*, ginsenoside Rb1 pretreatment attenuated tumor necrosis factor-*α*- (TNF-*α*-) induced oxidative stress, inflammation, and apoptosis in human umbilical vein endothelial cells [24]. Therefore, the antioxidant properties of ginseng contribute to important mechanisms that protect hepatic function [25]. Ginseng improves hepatic function by increasing the antioxidant enzyme activities of superoxide dismutase (SOD), catalase (CAT), and glutathione peroxidase (GPX) [26]. In previous experimental studies, administration of ginseng significantly increased glutathione peroxidase activity (GPX) in the liver, and, after exhaustive exercise, the GPX and SOD activities of the ginseng group rats were also significantly increased compared to those of the control rats [27]. In a clinical observation of antioxidant effects on healthy subjects, it was shown that ginseng significantly decreased malondialdehyde (MDA) and reactive oxygen species (ROS) levels, indicating that ginseng has antioxidant effects [28]. ROS is a by-product of oxygen metabolism, and the production of ROS plays an important role in hepatic dysfunction [29].

In addition, the anti-inflammatory effect of ginseng is another important factor in improving hepatic dysfunction. Most acute and chronic liver diseases are characterized by inflammatory processes. TNF-*α* plays an important role in inflammation by triggering the production of other cytokines that together recruit inflammatory cells, kill hepatocytes, and initiate a hepatic healing response that includes fibrogenesis [30]. *In vivo*, the acidic polysaccharide extract of ginseng effectively inhibits the production of proinflammatory cytokines, such as TNF-*α*, interleukin (IL)-1*β*, IL-6, interferon (IFN)-*γ*, IL-12, and IL-18, and has multiple immunomodulatory effects [31]. Previous studies have shown that the antifibrosis effect of a type of ginsenoside AD-2 extracted from ginseng on thioacetamide-induced liver injury in mice is related to the inflammatory factors (including TNF-*α*, IL-1*β*, caspase-1, and IL-6) associated with hepatic fibrosis [32]. Korean red ginseng water extract decreased the mRNA expression levels of interleukin-6 (IL-6), thymic stromal lymphopoietin (TSLP), and TNF-*α* in the 1-chloro-2,4-dinitrobenzene- (DNCB-) induced BALB/c mouse model which develops atopic dermatitis- (AD-) like lesions and alleviates AD-like inflammatory responses [33]. For liver fibrosis in rats induced by CCl_4_, ginseng extract inhibits liver inflammation by downregulating rat hepatic prostaglandin E_2_ and tissue inhibitor metalloproteinase-1 (TIMP-1) [34]. Therefore, the antioxidant and anti-inflammatory effects of GS-KG9 may improve hepatic function.

Clinical studies have shown that ginseng has few SAEs, and the most common AEs are diarrhea, hot flash, insomnia, and constipation [35]. No SAEs associated with GS-KG9 occurred in this study. Most of the reported AEs were mild, such as diarrhea, rash, and dyspepsia. There was no significant difference in the rate of AEs between the GS-KG9 and placebo groups (*p* > 0.05). This study shows that ginseng treatment can be considered safe.

This study has some limitations. First, the sample size was relatively small and the enrolled subjects were at the boundary between healthy people and patients. No chronic liver disease such as viral hepatitis or cirrhosis was observed, meaning that we cannot generalize our results to other hepatic disorders. Second, we only used liver enzymes to evaluate the effects of GS-KG9 on hepatic dysfunction and did not include computed tomography (CT) scans, MRI, or liver biopsies. These issues should be resolved in future research.

## 5. Conclusion

After the 12-week treatment, GS-KG9 significantly reduced ALT and GGT levels and had good safety results. The results of this study suggest that the GS-KG9 might be an effective and safe option for treating mild hepatic dysfunction. GS-KG9 may improve hepatic function through its antioxidant and anti-inflammatory effects.

## Figures and Tables

**Table 1:** Composition of test products provided.

Component	GS-KG9 (%)	Placebo (%)
*Panax ginseng* extract powder	80	0
Crystalline cellulose	18	90
Ginseng flavor powder	0	4
Caramel coloring	0	4
Magnesium stearate	1	1
Silicon dioxide	1	1
Total	100	100

GS-KG9: *Panax ginseng* extract.

**Table 2:** Baseline characteristics of the study subjects.

	GS-KG9 (*n* = 30)	Placebo (*n* = 30)	*p* value^1)^
Sex (M/F)	26/4	28/2	0.671^2)^
Age (years)	42.60 ± 11.74	41.73 ± 7.15	0.731
Height (cm)	171.83 ± 9.79	171.37 ± 7.03	0.833
Weight (kg)	83.64 ± 18.16	79.42 ± 11.35	0.287
BMI (kg/m^2^)	28.02 ± 3.95	26.91 ± 2.41	0.195
Waist circumference (cm)	93.86 ± 11.37	90.22 ± 8.13	0.158
SBP (mmHg)	120.47 ± 12.65	124.53 ± 16.06	0.280
DBP (mmHg)	78.77 ± 10.77	81.93 ± 10.40	0.251
Pulse (BPM)	75.60 ± 11.36	73.60 ± 8.89	0.451
Alcohol (*n*, %)	21 (70.00)	21 (70.00)	1.000^2)^
Alcohol (units/week)	8.64 ± 6.53	10.76 ± 6.88	0.312
Smoking (*n*, %)	9 (30.00)	11 (36.67)	0.584^2)^
Smoking (cigarettes/day)	14.00 ± 5.55	12.91 ± 4.91	0.646
ALT (IU/L)	64.67 ± 19.92	62.50 ± 20.84	0.682
GGT (IU/L)	67.17 ± 41.42	103.23 ± 171.92	0.272
AST (IU/L)	40.80 ± 13.96	41.17 ± 17.85	0.930
ALP (IU/L)	208.90 ± 50.37	220.90 ± 58.95	0.400
Total bilirubin (mg/dL)	0.71 ± 0.30	0.89 ± 0.36	0.043^*∗*^

Values are presented as mean ± standard deviation or number (%), ^1)^Analyzed by independent *t*-test; ^2)^analyzed by Fisher's exact test or Chi-square test; ^*∗*^*p* < 0.05. GS-KG9: *Panax ginseng* extract; BMI: body mass index; ALT: alanine transaminase; GGT: gamma-glutamyl transferase; AST: aspartate transaminase; ALP: alkaline phosphatase.

**Table 3:** Changes in hepatic function after 12 weeks of treatment.

	GS-KG9 (*n* = 26)		Placebo (*n* = 25)		*p* value^1)^	Adj.*p* value^2)^
	Baseline	12 weeks	Baseline	12 weeks		
ALT (IU/L)	64.77 ± 21.13	54.62 ± 23.09	59.28 ± 16.90	64.24 ± 25.40	0.009^*∗∗*^	0.009^*∗∗*^
GGT (IU/L)	64.85 ± 42.33	61.00 ± 40.00	62.00 ± 30.48	69.96 ± 42.21	0.038^*∗*^	0.036^*∗*^
AST (IU/L)	40.65 ± 14.93	34.81 ± 12.86	37.04 ± 10.24	36.20 ± 11.27	0.146	0.146
ALP (IU/L)	205.42 ± 48.76	201.81 ± 49.26	210.40 ± 52.81	214.72 ± 58.37	0.248	0.248
Total bilirubin (mg/dL)	0.72 ± 0.32	0.75 ± 0.29	0.86 ± 0.33	0.79 ± 0.25	0.261	0.211

Values are presented as mean ± standard deviation. ^1)^Analyzed by the independent *t-*test; ^2)^analyzed by ANCOVA (adjusted for total bilirubin); ^*∗∗*^*p* < 0.01; ^*∗*^*p* < 0.05. GS-KG9: *Panax ginseng* extract; ALT: alanine transaminase; GGT: gamma-glutamyl transferase; AST: aspartate transaminase; ALP: alkaline phosphatase.

**Table 4:** Changes in laboratory tests after 12 weeks of treatment.

	GS-KG9 (*n* = 30)		Placebo (*n* = 30)		*p* value^1)^
	Baseline	12 weeks	Baseline	12 weeks	
WBC (×10^3^/*μ*L)	6.62 ± 1.87	6.58 ± 1.64	6.88 ± 1.80	6.88 ± 1.86	0.924
RBC (×100^3^/*μ*L)	5.05 ± 0.44	4.99 ± 0.39	5.26 ± 0.49	5.19 ± 0.51	0.981
Hemoglobin (g/dL)	15.54 ± 1.32	15.39 ± 1.32	15.95 ± 1.24	15.76 ± 1.20	0.707
Hematocrit (%)	46.65 ± 3.90	45.85 ± 3.51	47.30 ± 3.45	46.46 ± 3.44	0.993
Platelet (×10^3^/*μ*L)	262.47 ± 75.47	258.14 ± 69.09	255.17 ± 41.34	253.23 ± 44.83	0.726
Total protein (g/dL)	7.28 ± 0.59	7.10 ± 0.53	6.99 ± 1.24	7.03 ± 0.39	0.329
Albumin (g/dL)	4.48 ± 0.37	4.33 ± 0.23	4.44 ± 0.38	4.30 ± 0.28	0.960
BUN (mg/dL)	13.46 ± 3.40	13.36 ± 3.26	12.80 ± 3.93	13.03 ± 3.86	0.614
Creatinine (mg/dL)	0.96 ± 0.17	0.97 ± 0.13	0.94 ± 0.12	0.93 ± 0.13	0.664
Glucose (mg/dL)	98.93 ± 12.27	102.63 ± 14.18	106.27 ± 30.94	107.03 ± 28.42	0.536
TC (mg/dL)	205.47 ± 36.39	206.53 ± 34.27	216.03 ± 32.12	218.40 ± 29.11	0.860
TG (mg/dL)	232.57 ± 175.44	182.27 ± 129.88	257.97 ± 250.73	222.37 ± 125.43	0.755
HDL-C (mg/dL)	46.23 ± 9.40	46.77 ± 8.81	45.60 ± 11.70	46.23 ± 10.24	0.958
LDL-C (mg/dL)	132.77 ± 37.09	140.23 ± 35.51	141.40 ± 38.21	142.00 ± 40.28	0.479
hs-CRP (mg/L)	1.47 ± 1.36	1.41 ± 1.16	1.63 ± 2.50	1.30 ± 1.22	0.522

Values are presented as mean ± standard deviation. ^1)^Analyzed by the independent *t*-test. GS-KG9: *Panax ginseng* extract; WBC: white blood cells; RBC: red blood cells; BUN: blood urea nitrogen; TC: total cholesterol; TG: triglyceride; HDL-C: high-density lipoprotein cholesterol; LDL-C: low-density lipoprotein cholesterol; hs-CRP: highly sensitive-C reactive protein.

## Data Availability

The datasets used and/or analyzed during the current study are available from the corresponding author upon reasonable request.
